# Removing entorhinal cortex input to the dentate gyrus does not impede low frequency oscillations, an EEG-biomarker of hippocampal epileptogenesis

**DOI:** 10.1038/srep25660

**Published:** 2016-05-10

**Authors:** Martin Meyer, Friederike Kienzler-Norwood, Sebastian Bauer, Felix Rosenow, Braxton A. Norwood

**Affiliations:** 1 Department of Neurology, Philipps University, Marburg Germany; 2 Epilepsy Center Frankfurt Rhine-Main, Department of Neurology, Göthe University, Frankfurt am Main, Germany

## Abstract

Following prolonged perforant pathway stimulation (PPS) in rats, a seizure-free “latent period” is observed that lasts around 3 weeks. During this time, aberrant neuronal activity occurs, which has been hypothesized to contribute to the generation of an “epileptic” network. This study was designed to 1) examine the pathological network activity that occurs in the dentate gyrus during the latent period, and 2) determine whether suppressing this activity by removing the main input to the dentate gyrus could stop or prolong epileptogenesis. Immediately following PPS, continuous video-EEG monitoring was used to record spontaneous neuronal activity and detect seizures. During the latent period, low frequency oscillations (LFOs), occurring at a rate of approximately 1 Hz, were detected in the dentate gyrus of all rats that developed epilepsy. LFO incidence was apparently random, but often decreased in the hour preceding a spontaneous seizure. Bilateral transection of the perforant pathway did not impact the incidence of hippocampal LFOs, the latency to epilepsy, or hippocampal neuropathology. Our main findings are: 1) LFOs are a reliable biomarker of hippocampal epileptogenesis, and 2) removing entorhinal cortex input to the hippocampus neither reduces the occurrence of LFOs nor has a demonstrable antiepileptogenic effect.

Epilepsy is a common neurological disorder that is characterized by recurrent, unprovoked seizures^1^. Temporal lobe epilepsy (TLE) is considered to be the most common of the epilepsy syndromes and is often refractory to treatment^2^. Although its prevalence is high, TLE is not well understood. A recent study suggests that etiology is unidentifiable for more than half of all patients^3^.

After a potentially epileptogenic brain injury, a so-called Initial Precipitating Incident (IPI), such as a traumatic brain injury or a febrile seizure, there is often a silent or “latent” period lasting months or years, during which seizures do not occur^4^. Although much is known about this period of epileptogenesis^5^,^6^,^7^, our understanding remains incomplete. With better knowledge of epileptogenic processes, the latent period may provide a window of opportunity in which to either prevent the development of epilepsy or at least reduce its severity^8^.

Although aberrant electrographic activity is a common finding in both epilepsy patients and animal models^9^,^10^,^11^,^12^,^13^, reliable EEG-detectable biomarkers of either epilepto- or ictogenesis are lacking. Such biomarkers could be useful in diagnosing epilepsy and localizing seizure foci, as well as developing novel therapies^14^,^15^,^16^.

During the course of a previous experiment, we noticed that perforant pathway stimulation (PPS)-based rat models of TLE exhibit spontaneous, large-amplitude electrographic activity in the dentate gyrus during the latent period. These spontaneous events did not appear to be fast ripples (FRs)^13^, but rather low frequency oscillations (LFOs) that were occasionally accompanied by granule cell population spikes^17^. These unprovoked waveforms sometimes had nearly identical morphology to those evoked by low-frequency PPS (Fig. 1). This observation, along with a study demonstrating that entorhinal cortex lesion can significantly antagonize amygdala kindling^18^, led us to hypothesize that a potential mechanism of epileptogenesis, at least in PPS-based animal models, is repeated, aberrant entorhinal cortex input to the dentate gyrus, which “kindles” the hippocampus, ultimately causing epilepsy^18^,^19^. Kindling is a phenomenon where repeated electrical or chemical stimulation over days or weeks, which is at first sub-convulsive, eventually provokes behavioral seizures^20^,^21^.

The present study was designed to characterize electrographic activity in the hippocampus during epileptogenesis and test our hypothesis. Immediately following 8 h PPS^22^, animals were continuously monitored with video-EEG using depth electrodes located in the dorsal dentate gyrus. In some animals, the perforant pathway was transected immediately following pro-epileptogenic PPS and the effects on LFOs and epileptogenesis were evaluated both electrophysiologically and histologically.

## Results

### Low frequency oscillations (LFOs) occur often in (pre-) epileptic hippocampus

**Figure 1:**
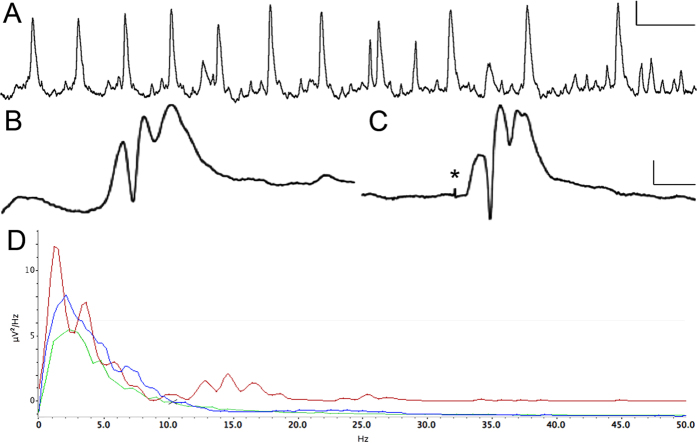
Spontaneous electrographic events recorded from the dentate gyrus in a freely-moving rat during the latent period following 8 hours of perforant pathway stimulation. (**A**) Twelve seconds of activity, demonstrating low frequency oscillations (LFOs) at a rate of 1 per second, with a frequency of 13.0 Hz. (**B**) Spontaneous unilateral EPSP with population spikes recorded from the granule cell layer. (**C**) Waveform evoked by 7.8 V perforant pathway stimulation. Note the high degree of similarity to panel **B**. All responses were obtained from the same rat three days post-stimulation. (**D**) Power Spectrum Density plot showing two minutes of LFOs (red), two minutes of baseline EEG in the same rat (blue), and two minutes of baseline EEG in non-epileptic control (green). Note the greater Power at 1 Hz and from 10–20 Hz, corresponding to the rate and frequency of LFO waveforms (red trace). Calibration bars = 1 s, 10 mV in A; 10 ms, 10 mV in B and C; 10 kHz sampling rate.

**Figure 2:**
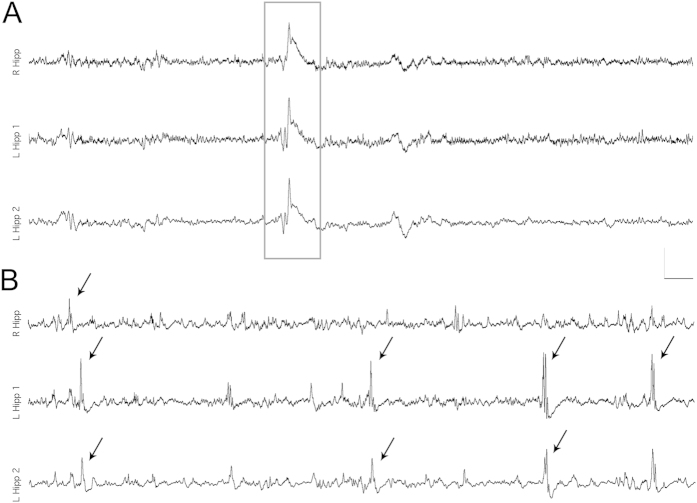
Synchronous and asynchronous low frequency oscillations (LFOs) recorded bilaterally from the dentate granule cell layer during the latent period. (**A**) A single LFO detected bilaterally. Note the high degree of synchrony within the left hippocampus as well as between hippocampi. (**B**) Nonsynchronous, bilateral LFOs. All events that exceeded the detection threshold are marked with arrows. Most, but not all, events that occurred in the left hippocampus were detected by both electrodes (L Hipp 1 and 2) which were separated by 2 mm laterally. All traces were obtained from the same freely moving rat twelve days post-stimulation, which was before the first spontaneous seizure. Calibration bars = 2 s, 5 mV; sampling rate 10 kHz.

**Figure 3:**
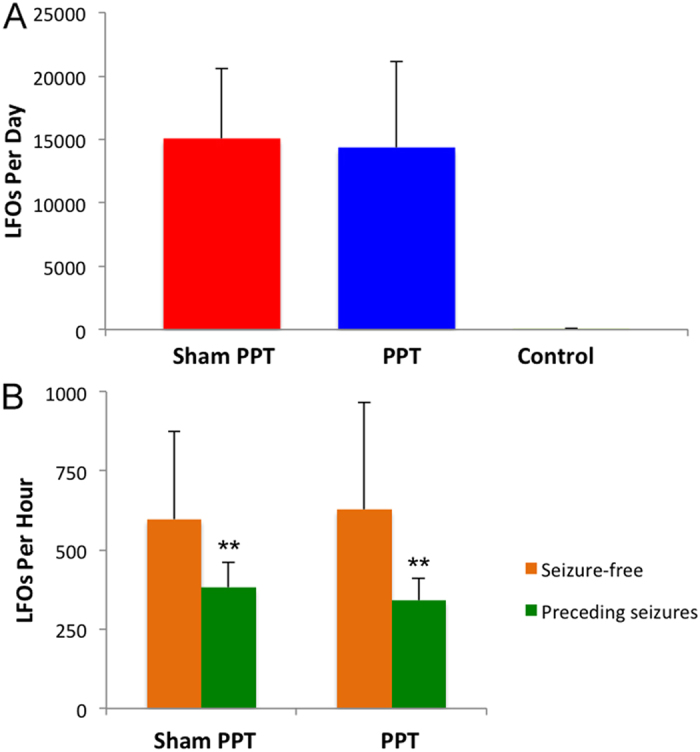
Low frequency oscillation (LFO) incidence in the dentate gyrus following 8 h perforant pathway stimulation (PPS). (**A**) The average number of LFOs detected per day during the latent period in sham perforant pathway transection (PPT) (8 h PPS, no PPT), PPT, and Control (8 h PPS, no PPT, did not develop epilepsy) (n = 4 per group). LFO occurrence was essentially the same in both Sham PPT and PPT groups (p > 0.05). No LFOs were detected in the Control group. (**B**) Average LFO occurrence per hour on seizure-free days (orange) and during the 60 minutes immediately prior to a spontaneous seizure (green). LFO occurrence usually decreased markedly in the hour preceding a spontaneous seizure (p < 0.001).

Continuous EEG recordings obtained from dentate granule cell layer revealed spontaneous events that began immediately following PPS (Fig. 1) and persisted until after the first spontaneous seizure. LFOs were detected both bilaterally and unilaterally (Fig. 2). Population spikes were present in fewer than 1% of LFOs and were always unilateral. Population spikes were often not detected by both electrodes in the same hippocampus (separated by 2 mm laterally). LFOs, with and without population spikes, occurred at a rate of one per second, had a period of approximately 0.067 seconds per cycle (Fig. 1), and occurred singly or in continuous periodic runs lasting an average of 33.8 ± 88.4 seconds. LFO amplitude (mV) varied by 1–3% during clusters. LFOs occurred randomly throughout the day and night at an average rate of 597.2 ± 276.8 per hour (Fig. 3). The rate per hour decreased markedly in the 60 minutes preceding a spontaneous seizure (382 ± 78.3 per hour, Fig. 3). No LFOs were detected in any rats prior to the 8 h stimulation or four stimulated rats that did not develop epilepsy.

### Perforant pathway transection (PPT) does not affect epileptogenesis

**Figure 4:**
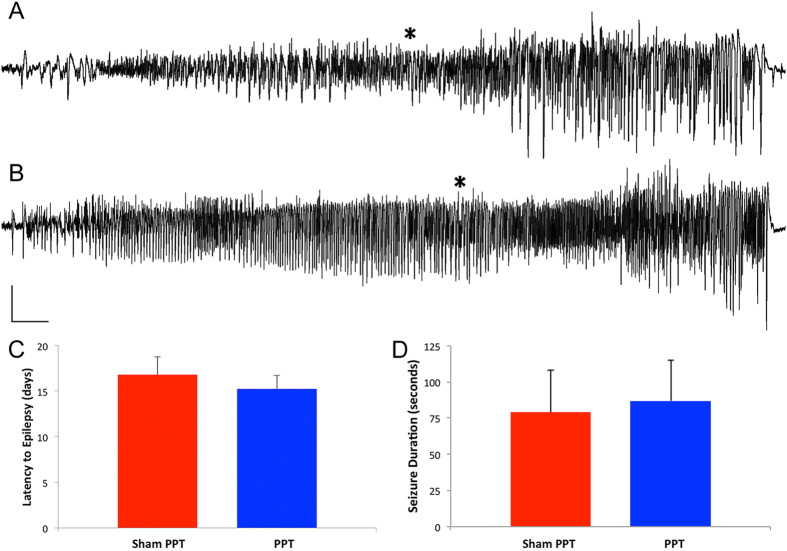
Spontaneous hippocampal-onset seizures observed in sham perforant pathway transection (Sham PPT) and PPT rats after 8 h of perforant pathway stimulation. Traces (**A**) (Sham PPT) and (**B**) (PPT) represent 65 seconds of activity recorded from the hippocampal granule cell layer in freely moving rats. Asterisks denote the first overt seizure behavior (forepaw clonus leading to rearing). (**C**) Mean latency to the first spontaneous seizure. The first seizures manifested after an average of 16.8 + 1.9 d (n = 6) in Sham PPT animals and 15.2 + 1.5 d in the PPT group (n = 5). (**D**) Mean spontaneous seizure duration. A total of twenty-six seizures were recorded from Sham PPT and PPT animals. Mean seizure length was 77 seconds for Sham PPT and 87 seconds for PPT rats. PPT had no affect on either the length of time between PPS and the first seizure (latent period) or seizure duration (p > 0.05). Calibration bars: 3 s, 4 mV.

Here we tested the hypothesis that removing entorhinal cortex input to the hippocampus alters epileptogenesis either by prolonging the latent period, i.e. duration to first spontaneous seizure, or reducing the severity of epilepsy, e.g. seizure duration. Continuous video-EEG monitoring revealed that bilateral PPT did not significantly alter the time between 8 h PPS and the first spontaneous seizure (Fig. 4). The average duration from 8 h PPS to the first spontaneous seizure was 16.8 d for the control group (Sham PPT, n = 6) vs 15.3 d (n = 4) for the PPT group (p > 0.05). Mean seizure length was 77 seconds for controls and 87 seconds for PPT rats (p > 0.05, Fig. 4) and all seizures were stage 4 or 5 on the Racine scale^23^.

### LFO occurrence is not impacted by PPT

Continuous monitoring of electrographic activity, with recording electrodes positioned in the hippocampal granule cell layer, was used to detect LFOs. LFO frequency was not significantly different in animals that received PPT (p > 0.05). An average of 14332.8 ± 5535.9 and 15045.2 ± 6796.7 LFOs were detected per day in Sham PPT and PPT groups, respectively (Fig. 3, n = 4 per group). A similar reduction in LFO frequency was seen in the hour preceding spontaneous seizures in both Sham PPT and PPT groups (36.1% and 45.2% reduction, respectively) (p > 0.05, Fig. 3).

### PPT does not alter the development of hippocampal sclerosis

In order to determine whether PPT, directly following pro-epileptogenic PPS, affects hippocampal atrophy, a characteristic of this animal model, we measured and compared hippocampal area in 21 rats that received 8 h of PPS with and without PPT.

Hippocampal area was essentially the same for both groups (p > 0.05), thereby demonstrating no effect of PPT on the development of hippocampal sclerosis. Mean values were 3.59 mm^2^ + 1.39 mm^2^ for Sham PPT (n = 8) and 3.20 mm^2^ + 1.09 mm^2^ for PPT rats (n = 13).

### Hippocampal neuron counting

**Figure 5:**
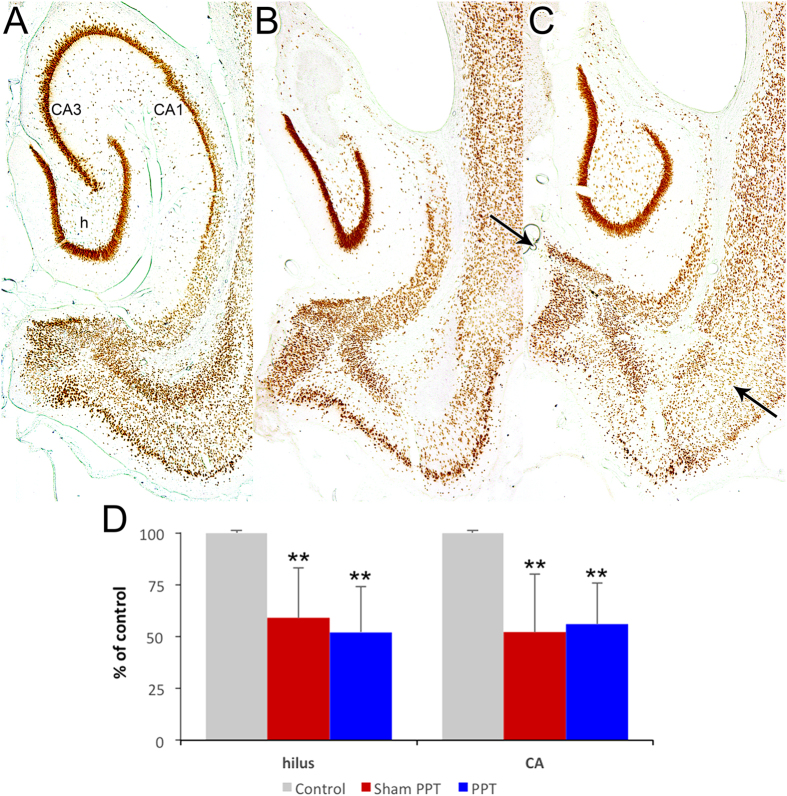
Hippocampal neuropathology ca. 2 months after 8 h perforant pathway stimulation in control (**A**), sham perforant pathway transection (Sham PPT, (**B**), and PPT (**C**) groups. NeuN-immunostained transverse sections demonstrating similar neuron loss in both experimental groups. Note the severe loss of CA3 and CA1 neurons in the hippocampus. Arrows in panel C denote the location of the mechanical lesion. (**D**) Quantification of hippocampal neuron loss. Neuron counts were performed on matching NeuN-immunostained sections. CA3 and CA1 were combined as the border between these regions was unclear in experimental animals. Although substantial neuron loss was seen in the hilus (h) and CA regions in both Sham PPT and PPT groups when compared with control (n = 5 per group, p < 0.001), there was no significant difference between groups (p > 0.05), demonstrating no effect of PPT on neurodegeneration.

To quantify the extent of neuron loss after 8 h perforant pathway stimulation, we analyzed ventral hippocampus sections obtained from untreated control, Sham PPT, and PPT groups (n = 5 per group) that were immunostained for NeuN^22^,^24^. The percentage of surviving hilar neurons was reduced by a similar extent in both Sham PPT and PPT groups (66% and 52% of control, respectively, p < 0.001), as was the percentage of surviving neurons in CA3/CA1 (52% and 56%, respectively, p < 0.001). There was, however, no significant difference between Sham PPT and PPT groups (p > 0.05), demonstrating no effect of PPT on hippocampal neurodegeneration (Fig. 5).

## Discussion

There are two principal, original findings in this study. First, that LFOs recorded from the dentate gyrus are a reliable biomarker of hippocampal epileptogenesis. Such events began immediately after 8 h PPS, occurred with seemingly no predictability, e.g. frequency, duration, time of day (p > 0.05 for all), were occasionally accompanied by population spikes, and persisted into chronic epilepsy. LFOs occurred at a rate of around one per second with a frequency of 13–17 Hz and a peak intensity of 15 Hz (Fig. 1). To the best of our knowledge, this is the first evidence implicating aberrant waveforms of this frequency with (experimental) epilepsy. Interestingly, the LFO rate (per hour) decreased significantly in the hour preceding a spontaneous seizure (Fig. 3). This could mean that LFOs function to maintain the inhibitory network and when their incidence decreases, the probability of a seizure increases. We, therefore, hypothesize that the rate of LFO occurrence is inversely proportional to likelihood of a spontaneous seizure.

The LFOs described in the present manuscript have not, to the best of our knowledge, been described in previous studies. The closest thing to LFOs was presented by White and colleagues^12^, but the frequency range of their “EEG spikes” was 0.15–0.6 Hz, much slower than LFOs, which are around 15 Hz. Furthermore, we found LFOs only in rats that developed epilepsy. White *et al.* found up to 2,000 such spikes/day in control animals^12^.

LFOs are similar to fast ripples (FRs), a high-frequency phenomenon described by Bragin and colleagues^9^, in that they are apparently collective depolarizations emanating from the hippocampus^13^, but in the present case seem to be localized to the ripening epileptic focus, rather than tissue adjacent to the lesion^17^. In fact, Bragin and colleagues stated that FRs do not emanate from the dentate gyrus, which is precisely where we detected LFOs^17^. Another important difference is that LFOs are much slower than FRs. LFOs occur approximately once per second with a periodicity of 0.067 seconds per cycle (corresponding to a frequency of 15 Hz), whereas FRs are on the order of 200–500 Hz^13^. Since FRs and LFOs have neither the same frequency nor the same origin, we postulate that the underlying mechanisms differ. Since LFOs exhibit similar morphology, i.e. large EPSP, to waveforms evoked by perforant pathway stimulation (Fig. 1) and EPSPs are the collective depolarization of granule cell dendrites^25^, we hypothesize that the likely source of LFOs is granule cell dendrites. Perhaps it is a collective, spontaneous depolarization of these dendrites that initiates LFOs and, conceivably, also spontaneous seizures.

Our second main finding is that removing entorhinal cortex input to the hippocampus after a pro-epileptic insult is not antiepileptogenic. This was done in an attempt to silence the aforementioned aberrant electrographic activity, i.e. LFOs, that we hypothesized to be epileptogenic. Transection of the perforant pathway, thereby interrupting the main fiber tract connecting the entorhinal cortex to hippocampus, did not prolong the latency to the first spontaneous seizure. Nor did PPT affect either seizure semiology or duration (p > 0.05, Fig. 4).

**Figure 6:**
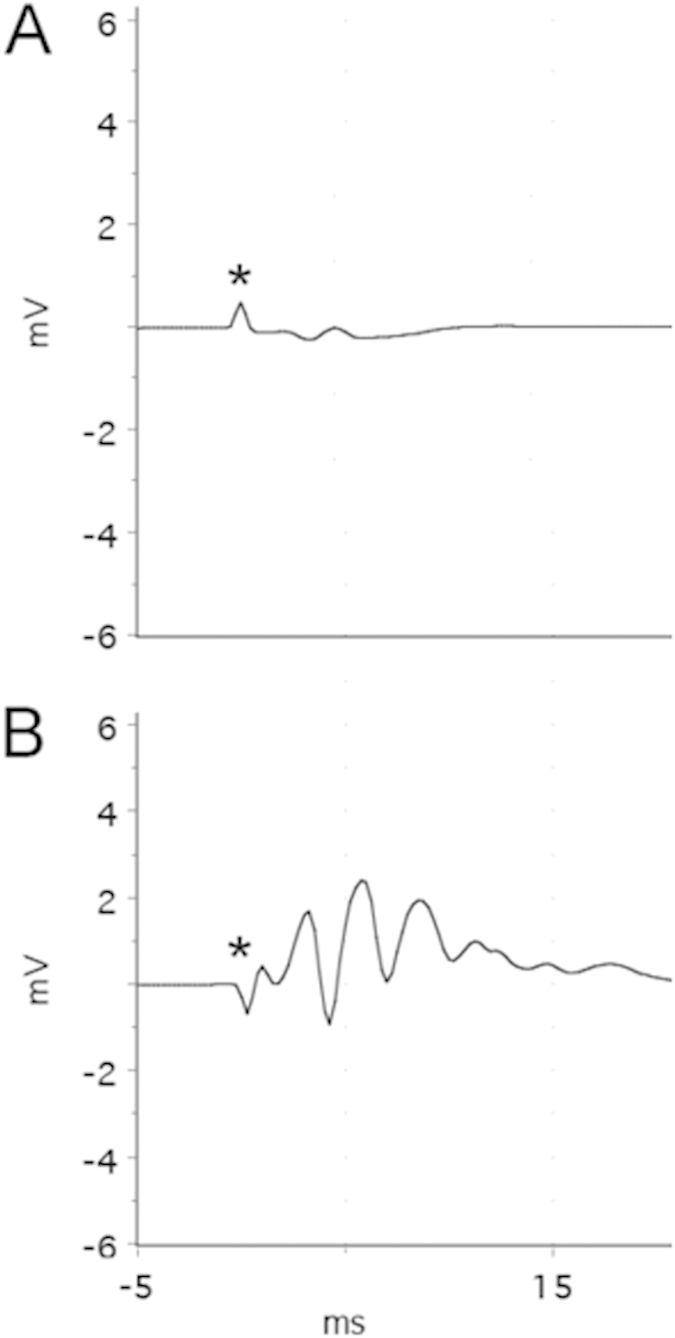
Electrophysiological confirmation of perforant pathway transection (PPT) efficacy *in vivo*. Representative field potentials recorded from the dentate gyrus, evoked by ipsilateral 0.2 Hz perforant pathway stimulation (PPS) at 20 V in (**A**): experimental (8 h PPS + PPT) and (**B**): control (8 h PPS + sham PPT) rats. The small waveform in panel A shows neither a large, positive wave (EPSP) nor population spikes, suggesting that PPT was effective. This is in contrast to the large, complex waveform seen in panel B, which is indicative of effective PPS, i.e. intact entorhinal cortex input to the dentate gyrus. Each panel represents the average of 10 responses.

There are limitations to these findings. First, PPT probably did not sever all connections between the entorhinal cortex and hippocampus. The projections connecting these structures are complex and the perforant pathway represents but the main conduit^26^. However the vast majority of fibers were cut, as evidenced by the inability of perforant pathway stimulation to evoke granule cell activity (Fig. 6). Post-lesion re-innervation (neuroplasticity) was also not studied. Steward *et al.* showed that after unilateral entorhinal cortex lesion the denervated DG is somewhat re-innervated by afferents from the contralateral EC after 9–16 days^27^, which is around the time that the first spontaneous seizures occur in this animal model^24^. Therefore, it is possible that (perhaps aberrant) nascent re-innervation plays a minor role in epileptogenesis, but studying this phenomenon was not a goal of this study and would require a different experimental design, e.g. a physical barrier to obstruct re-innervation.

An important caveat to lesion-based experiments is that both the type of insult and assays used can produce apparently different phenotypes. For example, remote spatial memory has been found to be impaired when the hippocampus is permanently lesioned^28^,^29^, but not when the hippocampus is temporarily and reversibly disrupted by lidocaine^30^. Reversible inactivation of the hippocampus with lidocaine apparently spares remote memory in the five-arm maze^31^, but not in the in the water maze^30^. It is important, therefore, to bear in mind that the interpretation of results can be influenced by experimental design.

An interesting approach would be to reversibly and specifically silence entorhinal cortex neurons with either optogenetics or Designer Receptors Exclusively Activated by Designer Drugs (DREADDs)^32^,^33^. Such methods would circumvent the need for a lesion (chemical or mechanical), be reversible, and target a particular cell population, e.g. Layer II of the entorhinal cortex or dentate granule cells. During epileptogenesis, a specific neuron type could be temporarily silenced and the resultant effect on hippocampal LFOs and epileptogenesis evaluated. Such an on-demand, precise treatment has potentially more therapeutic utility than a lesion, as it could be applied intermittently rather than permanently, thereby preserving normal function. It is known, for example, that permanent damage to the perforant pathway results in learning and memory impairment^34^,^35^.

In summary, 1) LFOs recorded from the dentate gyrus are a reliable biomarker of hippocampal epileptogenesis and 2) transecting the perforant pathway immediately after an epilepsy-inducing injury did not affect epileptogenesis in any of the measured electrophysiological or histological parameters. Therefore, we conclude that the direct EC-DG connection has a limited causal role in epileptogenesis and it is unlikely that a kindling effect, at least one induced by aberrant input from the EC, is important for either epileptogenesis or the development of epilepsy-associated neuroanatomical changes, e.g. hippocampal sclerosis.

## Methods

### Animal treatment

84 Male Sprague–Dawley rats (300–400 g; Harlan-Winkelmann, Borchen, Germany) were treated in accordance with the European Communities Council Directive (2010/63/EU). All experiments were approved by the local regulation authority (Regierungspräsidium Gieβen). Animals were housed in an on-site animal facility (21–25 °C; 31–47% humidity) under a 12:12 light/dark cycle and with ad libitum access to food and water.

### Bilateral electrode implantation

Under ketamine (120 mg/kg i.p.) and xylazine (5 mg/kg i.p.) anesthesia, bipolar stainless-steel stimulating electrodes (NEX-200, Rhodes Medical Instruments, Summerland, USA) were placed in the angular bundles of the perforant pathway and custom unipolar recording electrodes (crafted from 796000, A-M Systems, Carlsborg, USA) were lowered into each dorsal dentate gyrus. Electrode locations were determined by optimizing the potentials evoked by low-frequency PPS. Dental cement fixed the electrodes to anchor screws and the skull. Plastic connectors (GS09PLG, Ginder Scientific, Ottawa, Ontario, Canada) joined the electrodes with stimulation/recording equipment.

### Perforant pathway stimulation

All stimulation protocols utilized a paradigm designed to evoke and maintain hippocampal seizure activity throughout the stimulation, but not convulsive status epilepticus^4^,^24^,^36^. This consisted of continuous, bilateral 2 Hz paired-pulse stimuli, with a 40-ms interpulse interval, plus a 10-second train of 20 Hz single-pulse stimuli delivered once per minute, generated by a S88 stimulator (Grass Instruments, West Warwick, USA). All pulses (0.1 ms duration) were delivered at 20–24 V, as we have found this voltage to reliably evoke granule cell discharging without tissue-damaging hydrolysis^24^,^36^. The current associated with these voltages was typically between 15–30 μA. Stimulation evoked population spikes with an amplitude of 5–10 mV. No samples displayed any signs of hydrolysis, e.g. holes around the stimulating electrodes.

As described previously, stimulation for 30 minutes (on two consecutive days) required only isoflurane to terminate seizures^24^. Eight-hour stimulation on the third day did not induce status epilepticus and, therefore, did not require pharmacological termination. All animals survived the 8 h stimulation.

### Electrophysiological and video monitoring methods

EEG activity during and after PPS was amplified and recorded digitally at 10 kHz utilizing a PowerLab 16/35 and LabChart software versions 7 and 8 (AD Instruments, Mountain View, USA). Spontaneous granule cell layer activity was recorded continuously (24 h/d) and stored digitally and automatically in 3-hour epochs. Each day, the preceding 24 h of recordings were analyzed and all events with amplitudes exceeding 120% of average baseline (free of LFOs and seizures) were analyzed. Potential seizures were related to behavior on the time-stamped video recordings. Continuous (24/7) video monitoring utilized AirCam OD-325HD infrared cameras (Airlive Company, new Taipei city, Taiwan). Video files were captured at 15 frames/sec and time-stamped for integration with the electrophysiological data using SecuritySpy surveillance software (Ben Software, London, UK) and stored digitally. Confirmed seizures were scored according to the Racine scale^23^.

### LFO detection and analysis

As mentioned above, all EEG events with amplitude that exceeded threshold (120% of baseline amplitude) were analyzed. This was confirmed to include all spontaneous seizures as well as other events, such as LFOs. The Spike Histogram feature in LabChart v7 was used to quantify LFOs and measure their amplitude. The Spectrum feature, built-in to LabChart v8, generated Power Spectrum Density Plots using Welch’s method. Briefly, a Fast Fourier Transform size of 32 K (32768) was applied to the 10 kHz signal with a window overlap of 50% and the Power (uV^2^/Hz) of frequencies ranging from 0–50 Hz was plotted.

### Perforant pathway transection (PPT)

Immediately following the 8-h stimulation, animals were anesthetized with ketamine (120 mg/kg i.p.) and xylazine (5 mg/kg i.p.) and placed in a stereotaxic apparatus. Electrodes were removed and a crescent blade microknife (10317-14, FineScienceTools, Germany) was used to cut in the frontal plane between the entorhinal area and the hippocampus (bilaterally), which resulted in the destruction of the entorhinal afferents to the fascia dentata. The following coordinates measured from the interaural line were used with the knife angled backward against the frontal plane by 10°: AP 0; L 3 to 7; V down to the base of the skull^37^. Recording electrodes were re-implanted stereotactically in the dorsal dentate gyrus bilaterally using the same coordinates as the original recording electrodes. Control animals received treatment identical to the experimental group, with the only difference being a sham perforant pathway transection (bilateral trephination, but no knife insertion). The location and completeness of knife cuts were verified with low-frequency PPS immediately prior to sacrifice (Fig. 6) and histologically (Fig. 5).

### Perfusion-fixation and tissue treatment

At least two months after stimulation, in order to allow for completion of macroscopic anatomical changes, e.g. hippocampal sclerosis, rats were sacrificed in order to evaluate hippocampal neuropathology. Animals were deeply anesthetized with ketamine (200 mg/kg i.p.) and xylazine (5 mg/kg i.p.) and perfused through the aorta with 0.9% saline for 2 min followed by 10 min of 4% paraformaldehyde (0.1 M phosphate buffer, pH 7.4). Brains were immediately removed from the skull and placed in 4% paraformaldehyde. Following post-fixation at 4° for at least 48 h, 30-μm-thick transverse sections were cut on a freezing microtome. Free-floating sections were incubated overnight in primary mouse anti-NeuN (1:10,000 in phosphate buffered saline (PBS; MAB377; Chemicon, Temecula, USA) at room temperature. On the following day, sections were incubated in biotinylated secondary antibody solution (goat anti-mouse 1:2,000 in PBS; Vector, Burlingame, USA) for 2 h, washed twice in PBS, and then incubated for 2 h in avidin–biotin–HRP (horseradish peroxidase) complex (Vector Elite Kit 1:1,000 in PBS) at room temperature. Finally, sections were washed three times in PBS and developed in a solution containing hydrogen peroxide and diaminobenzidine.

### Hippocampal area quantification

Images of Nissl-stained sections were acquired with an Axio Imager M2 microscope (Zeiss, Göttingen, Germany). Equally spaced (every 5th section throughout the entire hippocampus), transverse sections were analyzed at 1.6× magnification. The area of the hippocampus was calculated for each section with Adobe Photoshop CS5 (Adope, San Jose, USA)^24^.

### Hippocampal neuron counting

To estimate the extent of neuron loss after 8 h PPS, we analyzed ventral hippocampus sections obtained from untreated control, Sham PPT, and PPT animals that were immunostained for NeuN^22^,^24^. Matching sections were selected, based on the identification of extrahippocampal brain regions, e.g., thalamic nuclei and ventricle shape. Cell counts included all immunopositive neurons with somata that contained a visible nucleus. In the hilus, this included cell bodies not in contact with the granule cell layers and outside the somal and dendritic regions of CA3c. In the untreated control sections, immunoreactive neurons in the pyramidal cell layers included somata that contacted at least one other neuron. Therefore, only neurons that were part of the pyramidal cell layer were counted. In sections from animals with hippocampal atrophy, in which strata boundaries were unclear, all immunoreactive neurons within the remaining hippocampus, but located outside the dentate gyrus, were counted. Cell counts did not use Nissl-stained sections because neurons could not be reliably differentiated from the many glial cells that proliferate after seizure-induced damage.

### Statistical analysis

Statistical analyses were performed using BIAS version 10.12 (epsilon-Verlag GbR, Darmstadt, Germany). Statistical disparity between groups was tested using the Wilcoxon-Mann-Whitney-Test. Histograms and calculations were created with Excel for Mac 2011 (Microsoft Corporation, Redmond, USA). All data are presented as mean ± standard deviation.

## Additional Information

**How to cite this article**: Meyer, M. *et al.* Removing entorhinal cortex input to the dentate gyrus does not impede low frequency oscillations, an EEG-biomarker of hippocampal epileptogenesis. *Sci. Rep.* **6**, 25660; doi: 10.1038/srep25660 (2016).
